# Racial differences in inflammation and outcomes of aging among kidney transplant candidates

**DOI:** 10.1186/s12882-019-1360-8

**Published:** 2019-05-17

**Authors:** Prakriti Shrestha, Christine E. Haugen, Nadia M. Chu, Ashton Shaffer, Jacqueline Garonzik-Wang, Silas P. Norman, Jeremy D. Walston, Dorry L. Segev, Mara A. McAdams-DeMarco

**Affiliations:** 1 Department of Surgery, Johns Hopkins University School of Medicine, Baltimore, MD USA; 2 Department of Epidemiology, Johns Hopkins School of Public Health, 615, N. Wolfe St, W6033, Baltimore, MD 21205 USA; 3 Department of Medicine, Division of Nephrology, University of Michigan, Ann Arbor, MI USA; 4 Department of Medicine, Division of Geriatrics, Johns Hopkins University School of Medicine, Baltimore, MD USA

**Keywords:** End-stage renal disease, Inflammation, Race, Frailty, HRQOL

## Abstract

**Background:**

Inflammation is more common among African Americans (AAs), and it is associated with frailty, poor physical performance, and mortality in community-dwelling older adults. Given the elevated inflammation levels among end-stage renal disease (ESRD) patients, inflammation may be associated with adverse health outcomes such as frailty, physical impairment, and poor health-related quality of life (HRQOL), and these associations may differ between AA and non-AA ESRD patients.

**Methods:**

One thousand three ESRD participants were recruited at kidney transplant evaluation (4/2014–5/2017), and inflammatory markers (interleukin-6 [IL-6], tumor necrosis factor-a receptor-1 [TNFR1], C-reactive protein [CRP]) were measured. We quantified the association with frailty (Fried phenotype), physical impairment (Short Physical Performance Battery [SPPB]), and fair/poor HRQOL at evaluation using adjusted modified Poisson regression and tested whether these associations differed by race (AA vs. non-AA).

**Results:**

Non-AAs had lower levels of TNFR1 (9.7 ng/ml vs 14.0 ng/ml, *p* < 0.001) and inflammatory index (6.7 vs 6.8, *p* < 0.001) compared to AAs, but similar levels of IL-6 (4.5 pg/ml vs 4.3 pg/ml, *p* > 0.9) and CRP (4.7 μg/ml vs 4.9 μg/ml, *p* = 0.4). Non-AAs had an increased risk of frailty with elevated IL-6 (RR = 1.58, 95% CI:1.27–1.96, *p* < 0.001), TNFR1 (RR = 1.60, 95% CI:1.25–2.05, *p* < 0.001), CRP (RR = 1.41, 95% CI:1.10–1.82, *p* < 0.01), and inflammatory index (RR = 1.82, 95% CI:1.44–2.31, *p* < 0.001). The associations between elevated inflammatory markers and frailty were not present among AAs. Similar results were seen with SPPB impairment and poor/fair HRQOL.

**Conclusions:**

Non-AAs with elevated inflammatory markers may need closer follow-up and may benefit from prehabilitation to improve physical function, reduce frailty burden, and improve quality of life prior to transplant.

## Background

ESRD patients experience accelerated aging resulting in adverse health outcomes of aging such as frailty, lower extremity impairment, and poor health-related quality of life (HRQOL) [1–5]. More specifically, frailty, a syndrome of decreased physiologic reserve [6], is present in 41.8% of hemodialysis patients [2, 3, 7] and is associated with falls [2], hospitalizations [1, 7, 8], poor cognitive function [3], decreased HRQOL [4], and mortality [1, 7]. Lower extremity impairment, measured using the Short Physical Performance Battery (SPPB) [9], is an important predictor of mortality in ESRD patients undergoing KT and is associated with longer length of stay after KT [5]. Additionally, poor HRQOL is associated with frailty [4, 10] and predicts cardiovascular health and physical performance in ESRD patients [11].

In community dwelling older adults, elevated inflammatory markers including interleukin 6 (IL-6), C-reactive protein (CRP), and tumor necrosis factor-α receptor-1 (TNFR1) are associated with higher risk of multiple adverse health outcomes of aging including frailty [12], disability [13], and decreased grip strength [14]; furthermore, these adverse health outcomes of aging are important risk factors for mortality [15, 16]. Additionally, chronic dialysis is associated increased inflammation [17], and inflammation is a risk factor for mortality in ESRD patients [18, 19]. Among patients undergoing hemodialysis, there is a differential impact of inflammatory markers on mortality by race [20, 21]. Therefore, it is likely that the association between inflammatory marker levels and adverse health outcomes of aging also differ by race.

Quantifying the association between inflammatory markers and adverse health outcomes of aging such as frailty, lower extremity impairment, and poor HRQOL may provide insight into the role of aging biology to explain racial disparities related to poor outcomes for ESRD patients. Thus, the goals of this study were to: 1) quantify the association between inflammation and adverse health outcomes of aging (frailty, lower extremity function, and poor HRQOL) and 2) test whether there are differential associations between elevated inflammatory markers and adverse health outcomes of aging by race (African American [AA] vs. non-AA) among KT candidates.

## Methods

### Study design

This was a cross-sectional cohort study of 1003 English-speaking ESRD consecutive participants 18 years or older who were evaluated for KT at the Johns Hopkins Hospital, Baltimore, Maryland, from April 2014 to May 2017 (*N* = 891) and the University of Michigan, Ann Arbor, Michigan, from July 2015 to March 2016 (*N* = 112). Participants were eligible for enrollment if they were English speaking and aged 18 or older; the refusal rate during the enrollment period was 18%. At the time of KT evaluation, we measured Fried frailty, SPPB, HRQOL, and obtained a blood sample as described below. Additional participant characteristics were also assessed at KT evaluation or abstracted from the transplant evaluation medical record (age, sex, race, time on dialysis, type of dialysis, Charlson comorbidity index [CCI]). Race was measured using self-reported data from the electronic health record, EPIC. Participants selected among Black/African American (AA), White/Caucasian, Asian, Native Hawaiian, or Other. Participants who were not on dialysis at time of KT evaluation were classified as pre-emptive transplant. Comorbidities were classified as present if the participant currently suffered from the disease or had a previous history the disease. The Johns Hopkins Institutional Review Board and the University of Michigan Institutional Review Board approved the study and participants provided written informed consent.

### Frailty

We studied the physical frailty phenotype defined and validated by in community-dwelling older adults [6, 10, 22–30] and by our research group in ESRD and KT populations [2–5, 7, 8, 31–38]. Frailty was defined as a score of three or more of the Fried frailty components: shrinking (self-report of unintentional weight loss of more than 10 pounds in the past year based on dry weight); weakness (grip-strength below an established cutoff based on gender and BMI); exhaustion (self-report); low activity (Kcals/week below an established cutoff); and slowed walking speed (walking time of 15 ft below an established cutoff by gender and height) [6]. Each of the 5 components was scored as 0 (absence) or 1 (presence). The aggregate frailty score was calculated as the sum of the component scores (range 0–5). Nonfrail was defined as a score of 0 and frail was defined as a score of ≥3.

### Short physical performance battery (SPPB)

The SPPB is an objective test of lower extremity function (balance, walking speed, repeated chair stands) [9]. Each component has a score ranging from 0 to 4, for a summed composite score ranging from 0 to 12. SPPB impairment was defined as having a SPPB score < 10 on a scale 0 to 12 [5, 9]. The test is administered by trained research assistants and takes approximately 5 min to complete. For the balance portion, recipients are asked to stand and remain in several progressively more difficult positions (side-by-side, semi-tandem, and full-tandem stances) for 10 s each. For the walking speed test, recipients’ walking speed is measured as they are asked to walk 15-ft at a normal pace. Finally, for the chair stand portion, recipients are asked to fold their arms across their chest and rise from a chair five times as quickly as possible. A full description of administration and scoring has been detailed elsewhere [9].

### Health related quality of life (HRQOL)

HRQOL was measured using a single question instrument for global subjective health from the kidney disease quality of life assessment: “In general, would you say your health is …”. Participants reported HRQOL as being “Excellent”, “Very Good”, “Good”, “Fair” or “Poor” during KT evaluation. These classifications have been used in previous populations of ESRD patients and KT recipients [4, 37].

### Measures of inflammation

Serum inflammatory markers, IL-6, TNFR1, and CRP, were collected at KT evaluation, as these markers are frequently elevated in frailty and end-stage renal disease [38]. The blood samples were collected after evaluation in kidney transplant clinic at the same time as routine kidney transplant lab testing for all participants. There were no differences in sample volume (10 mL) between hemodialysis and non-hemodialysis participants. A full description of inflammation measurements performed by our research group has been detailed elsewhere [38]. We calculated the inflammatory index score using IL-6 and TNFR1 as has been previously published: [1/3 x log(IL-6)] + [2/3 x log(TNFR1)] [16]. Elevated inflammation markers were defined as >1SD higher level of IL-6, TNFR1, CRP or inflammation index on the log scale [38].

### Adverse health outcomes of aging and inflammation

Modified Poisson regression was used to estimate the association between inflammatory markers (IL-6, TNFR1, CRP, and inflammatory index) and adverse health outcomes of aging (frailty, SPPB impairment, and poor HRQOL). Elevated inflammation markers were defined as >1SD higher level of IL-6, TNFR1, CRP or inflammation index on the log scale. For each change in 1SD, there was an association between inflammation an adverse health outcome of aging. These models were stratified by race (AA and non-AA) and adjusted for age, sex, CCI, type of dialysis (hemodialysis, peritoneal dialysis, and no dialysis), and time on dialysis. We also used modified Poisson regression with a Wald test to estimate the interaction between non-AA and AA for each inflammatory marker with adjustment for the same confounders above.

### Statistical analysis

Continuous variables were compared using t-tests and categorical variables were compared using χ^2^ tests. For all analyses, a *P* value < 0.05 was considered significant. All analyses were performed using Stata 14.0 (College Station, Texas).

## Results

### Study population

Among 1003 KT evaluation participants, the mean age was 55 (SD = 13), 40.0% were female, 41.3% were AA, 53.0% were on hemodialysis, and 34.4% were evaluated for pre-emptive KT (Table 1). The mean CCI score was 2.0 (SD = 2.4) (Table 1). AA KT candidates were more likely to be younger (mean age at evaluation: 53 years vs. 56 years, *p* < 0.001), have diabetes (49.2% vs. 40.7%, *p* = 0.008), and be HIV+ (8.7% vs. 0.9%, p < 0.001) (Table 1). The mean CCI score (2.1 vs. 2.0, *p* = 0.50) was similar between AAs and non-AAs. AAs were more likely to be on hemodialysis (61.9% vs. 45.1%, *p* < 0.001) and more likely to be on dialysis for more than 2 years (32.4% vs. 19.4%, *p* < 0.001), but less likely to evaluated for pre-emptive transplant (26.2% vs. 43.2%, *p* < 0.001).

**Table 1:** Characteristics of kidney transplant (KT) candidates by race

Characteristics	Non-African American	African American	*p* value
	*n* = 589, 58.7%	*n* = 414, 41.3%	
Age, years^a^	56.2 (13.4)	52.9 (12.9)	< 0.001
Female, %	36.0	38.4	0.44
Charlson comorbidity index^a^	2.0 (2.5)	2.1 (2.2)	0.50
Comorbidities, %			
Myocardial infarction	12.1	9.9	0.28
Peripheral vascular disease	9.0	6.0	0.09
Cerebral vascular disease	8.0	9.0	0.59
Dementia	0.9	0.7	0.83
Chronic lung disease	4.5	3.4	0.41
Rheumatologic disease	5.5	8.1	0.10
Peptic ulcer disease	3.8	2.2	0.16
Diabetes	40.7	49.2	0.008
Diabetes with complications	26.2	26.9	0.82
Moderate/severe liver disease	3.3	5.6	0.07
Metastatic cancer	0.9	0.7	0.82
Leukemia	0.9	0.2	0.22
Lymphoma	1.2	0.5	0.24
Congestive heart failure	14.3	14.3	1.00
HIV+	0.9	8.7	< 0.001
Dialysis type			< 0.001
No dialysis	43.2	26.2	
Hemodialysis	45.1	61.9	
Peritoneal dialysis	11.8	11.9	
Time on dialysis			< 0.001
0 year	48.2	29.0	
1 or 2 years	32.4	38.6	
More than 2 years	19.4	32.4	
Inflammatory Marker^b^			
IL-6, pg/ml	4.5 [2.8–8.4]	4.3 [2.8–8.1]	0.95
TNFR1, ng/ml	9.7 [5.8–17.7]	14.0 [7.8–20.5]	< 0.001
CRP, ug/ml	4.7 [1.9–10.2]	4.9 [2.2–11.0]	0.36
Inflammatory Index	6.7 [6.2–7.1]	6.8 [6.5–7.2]	< 0.001
Frail status, %			0.01
Nonfrail	55.2	48.3	
Intermediate Frail	28.7	28.3	
Frail	16.1	23.4	
SPPB impairment, %	36.3	46.0	0.003
Fair/Poor HRQOL, %	48.0	50.9	0.37

^a^mean (standard deviation)

^b^median [IQR]

*SPPB* Short Physical Performance Battery, *HRQOL* Health Related Quality of Life, *IL-6* Interleukin-6, *TNFR1* tumor necrosis factor-α receptor-1, *CRP* C-reactive protein

The aggregate inflammatory index consists of IL-6 and TNFR1

### Race and inflammatory markers

**Fig. 1:**
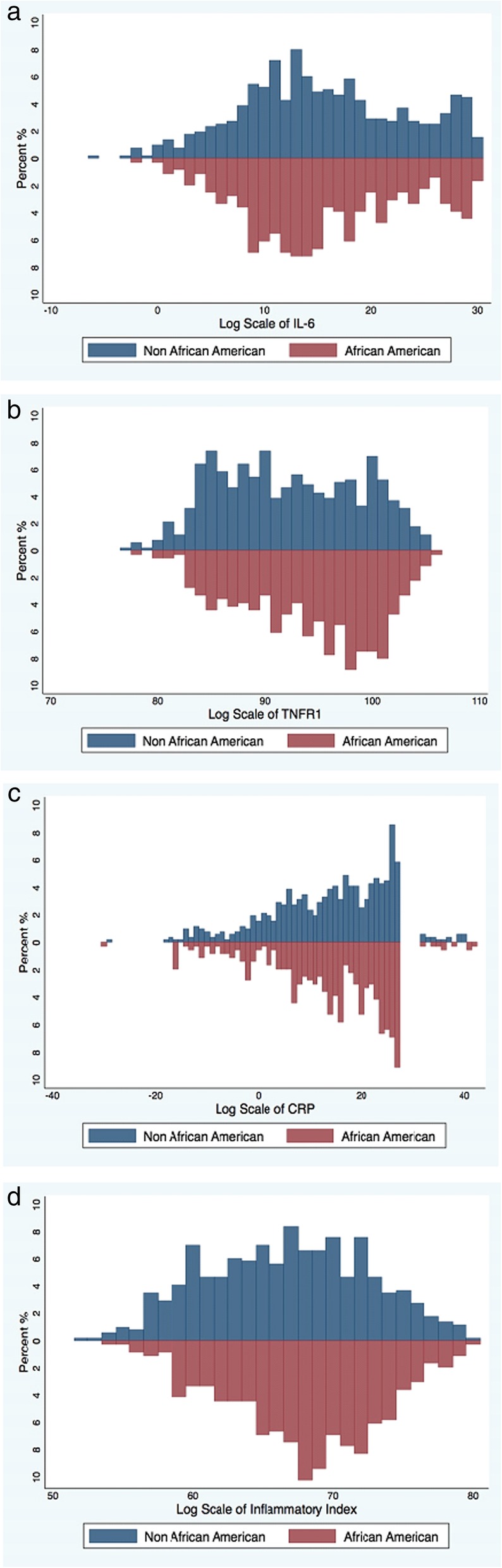
Distribution of inflammatory markers [(**a**) IL-6 (*p* > 0.9), (**b**) TNFR1 (*p* < 0.001), (**c**) CRP (*p* = 0.4), (**d**) inflammation index (*p* < 0.001)] by race (Non-African American vs. African American) among 1003 end-stage renal disease participants. The inflammatory index score was calculated using IL-6 and TNFR1: [1/3 x log(IL-6)] + [2/3 x log(TNFR1)]

AAs had higher TNFR1 levels (median [IQR]: 14.0 [7.8–20.5] ng/ml vs. 9.7 [5.8–17.7] ng/ml, p < 0.001) and inflammatory index scores (6.8 [6.5–7.2] vs. 6.7 [6.2–7.1], p < 0.001) compared to non-AAs (Table 1) (Fig. 1a, d). However, AAs had similar IL-6 (4.3 [2.8–8.1] pg/ml vs. 4.5 [2.8–8.4] pg/ml, *p* = 0.95) and CRP (4.9 [2.2–11.0] ug/ml vs. 4.7 [1.9–10.2] ug/ml, *p* = 0.36) levels compared to non-AAs (Table 1, Fig. 1b, c).

### Race and adverse health outcomes of aging

At the time of evaluation, 19.1% of KT candidates were frail, 40.0% had SPPB impairment, and 49.2% reported poor HRQOL. Additionally, AAs were more likely to be frail (23.4% vs. 16.1%, *p* = 0.01) and have SPPB impairment (46.0% vs. 36.3%, *p* = 0.003) compared to non-AAs, but the prevalence of poor HRQOL was similar between AAs and non-AAs (50.9% vs. 48.0%, *p* = 0.37) (Table 1).

### Frailty, inflammation, and race

The risk of frailty associated with elevated IL-6 varied by participant race (interaction *p* = 0.04): Elevated IL-6 was associated with a 1.58-fold (95% CI: 1.27–1.96, *p* < 0.001) increased risk of frailty among non-AAs but did not increase the risk of frailty among AAs (aRR: 1.18, 95% CI: 0.99–1.42, *p* = 0.06) (Table 2). The risk of frailty associated with elevated TNFR1 also varied by participant race (interaction *p* = 0.02): Elevated TNFR1 was associated with a 1.60-fold (95% CI: 1.25–2.05, *p* < 0.001) increased risk of frailty among non-AAs, but it was not associated with an increased risk of frailty among AAs (aRR: 1.18, 95% CI: 0.92–1.51, *p* = 0.2). The risk of frailty by elevated CRP did not vary by participant race (interaction *p* = 0.1): Elevated CRP was associated with a 1.41-fold (95% CI: 1.10–1.82, *p* < 0.01) increased risk of frailty among non-AAs but not among AAs (aRR: 1.11, 95% CI: 0.92–1.33, *p* = 0.3). The risk of frailty associated with elevated inflammatory index varied by participant race (interaction *p* < 0.001): Elevated inflammatory index was associated with a 1.82-fold (95% CI: 1.44–2.31, *p* < 0.001) increased risk of frailty among non-AAs and a 1.26-fold (95% CI: 1.02–1.56, *p* = 0.03) increased risk of frailty among AAs (Table 2).

**Table 2:** Association between inflammatory markers and adverse health outcomes of aging (frailty, SPPB impairment, and poor HRQOL) by race

Outcome	Inflammatory Marker	Race	RR (95% CI)	Interaction *p* value	aRR (95% CI)	Interaction *p* value
Frailty	IL-6	Non-AA	1.63 (1.36, 1.94)	0.05	1.58 (1.27, 1.96)	0.04
		AA	1.27 (1.08, 1.51)		1.18 (0.99, 1.41)	
	TNFR1	Non-AA	1.39 (1.19, 1.62)	0.02	1.60 (1.25, 2.05)	0.02
		AA	1.04 (0.87, 1.25)		1.18 (0.92, 1.51)	
	CRP	Non-AA	1.44 (1.15, 1.81)	0.08	1.41 (1.10, 1.82)	0.1
		AA	1.11 (0.91, 1.37)		1.11 (0.92, 1.33)	
	Inf. Index	Non-AA	1.60 (1.35, 1.89)	0.01	1.82 (1.44, 2.31)	< 0.01
		AA	1.17 (0.97, 1.40)		1.26 (1.02, 1.56)	
SPPB impairment	IL-6	Non-AA	1.56 (1.41, 1.72)	0.01	1.49 (1.33, 1.67)	< 0.01
		AA	1.28 (1.15, 1.42)		1.20 (1.08, 1.32)	
	TNFR1	Non-AA	1.26 (1.14, 1.40)	0.03	1.47 (1.27, 1.71)	< 0.01
		AA	1.07 (0.95, 1.20)		1.17 (1.01, 1.36)	
	CRP	Non-AA	1.35 (1.21, 1.53)	0.03	1.32 (1.16, 1.49)	0.09
		AA	1.13 (1.01, 1.27)		1.14 (1.02, 1.27)	
	Inf. Index	Non-AA	1.45 (1.31, 1.61)	0.01	1.67 (1.48, 1.90)	< 0.001
		AA	1.19 (1.06, 1.34)		1.28 (1.13, 1.46)	
Fair/Poor HRQOL	IL-6	Non-AA	1.16 (1.07, 1.27)	0.01	1.19 (1.09, 1.29)	< 0.001
		AA	0.99 (0.89, 1.09)		0.96 (0.87, 1.06)	
	TNFR1	Non-AA	1.02 (0.94, 1.11)	< 0.01	1.06 (0.95, 1.19)	< 0.01
		AA	0.84 (0.77, 0.93)		0.88 (0.78, 1.00)	
	CRP	Non-AA	1.16 (1.06, 1.27)	0.01	1.18 (1.08, 1.30)	< 0.01
		AA	0.98 (0.89, 1.07)		0.99 (0.91, 1.09)	
	Inf. Index	Non-AA	1.09 (1.00, 1.18)	0.001	1.16 (1.05, 1.29)	< 0.001
		AA	0.87 (0.78, 0.96)		0.92 (0.82, 1.03)	

Modified Poisson regression models were adjusted for age, sex, CCI, dialysis type, and time on dialysis/dialysis vintage. Interaction p value marks the difference between Non-African American (non-AA) and African American (AA) participants

*SPPB* Short Physical Performance Battery, *HRQOL* Health Related Quality of Life, *IL-6* Interleukin-6, *TNFR1* tumor necrosis factor-α receptor-1, *CRP* C-reactive protein, *aRR* adjusted relative risk RR: relative risk

The aggregate inflammatory index consists of IL-6 and TNFR1

### SPPB impairment, inflammation, and race

The risk of SPPB impairment associated with elevated IL-6 varied by participant race (interaction *p* < 0.01): Elevated IL-6 was associated with a 1.49-fold (95%CI: 1.33–1.67, *p* < 0.001) increased risk of SPPB impairment among non-AAs and a 1.20-fold (95%CI: 1.08–1.32, *p* = 0.001) increased risk of SPPB impairment among AAs (Table 2). The risk of SPPB impairment associated with elevated TNFR1 also varied by participant race (interaction *p* < 0.01): Elevated TNFR1 was associated with a 1.47-fold (95% CI: 1.27–1.71, *p* < 0.001) increased risk of SPPB impairment among non-AAs and a 1.17-fold (95% CI: 1.01–1.36, *p* = 0.04) increased risk of SPPB impairment among AAs. The risk of SPPB impairment associated with elevated CRP did not vary by participant race (interaction *p* = 0.09): Elevated CRP was associated with an increased risk of SPPB impairment in non-AAs (aRR: 1.32, 95% CI: 1.16–1.49, *p* < 0.001) and AAs (aRR: 1.14, 95% CI: 1.02–1.27, *p* = 0.02). The risk of SPPB impairment associated with elevated inflammatory index varied by participant race (interaction *p* < 0.001): Elevated inflammatory index was associated with a 1.67-fold (95%CI: 1.48–1.90, *p* < 0.001) increased risk of SPPB impairment among non-AAs and a 1.28-fold (95%CI: 1.13–1.46, *p* < 0.001) increased risk of SPPB impairment among AAs (interaction *p* < 0.001) (Table 2).

### Fair/poor HRQOL, inflammation, and race

The risk of fair/poor HRQOL and elevated IL-6 varied by participant race (interaction *p* < 0.001): Elevated IL-6 was associated with a 1.19-fold (95% CI: 1.09–1.29, *p* < 0.001) increased risk of fair/poor HRQOL among non-AAs, but it was not associated an increased risk of fair/poor HRQOL among AAs (aRR: 0.96, 95% CI: 0.87–1.06, *p* = 0.4) (Table 2). The risk of having fair/poor HRQOL and elevated TNFR1 varied by participant race (interaction *p* < 0.01): Elevated TNFR1 was not associated with an increased risk of fair/poor HRQOL among non-AAs (aRR: 1.06, 95% CI: 0.95–1.19, *p* = 0.3) and AAs (aRR: 0.88, 95% CI: 0.78–1.00, *p* = 0.05). The risk of Fair/Poor HRQOL and elevated CRP varied by participant race (interaction *p* < 0.01): Elevated CRP was associated with a 1.18-fold (95% CI: 1.08–1.30, *p* < 0.001) increased risk of fair/poor HRQOL among non-AAs but was not associated with an increased risk of fair/poor HRQOL among AAs (aRR: 0.99, 95% CI: 0.91–1.09, *p* = 0.9). The risk of fair/poor HRQOL and elevated inflammatory index varied by participant race (interaction *p* < 0.001): Elevated inflammatory index was associated with a 1.16-fold (95% CI: 1.05–1.29, *p* < 0.01) increased risk of having fair/poor HRQOL among non-AAs, but it was not associated with an increased risk of fair/poor HRQOL among AAs (aRR: 0.92, 95% CI: 0.82–1.03, *p* = 0.1) (Table 2).

## Discussion

Using a two-center prospective cohort of 1003 ESRD participants evaluated for KT, we found that AAs had higher levels of TNFR1 (*p* < 0.001) and inflammatory index (*p* < 0.001) compared to non-AAs, and we found that AAs and non-AAs had similar levels of IL-6 (*p* > 0.9) and CRP (*p* = 0.4). Despite higher levels of some inflammatory markers in AAs, non-AAs had a higher risk of adverse health outcomes of aging, including frailty, SPPB impairment, and fair/poor HRQOL compared to AAs. In non-AAs elevation of all inflammatory markers (IL-6, TNFR1, CRP, and inflammatory index) were independently associated with an increased risk of frailty and SPPB impairment after accounting for potential confounders. Elevated inflammatory markers (IL-6, CRP, and inflammatory index) were associated with an increased risk of fair/poor HRQOL in non-AA KT candidates. Among AA KT candidates, elevated inflammatory markers (IL-6, TNFR1, CRP, and inflammatory index) were associated with an increased risk of SPPB impairment, and an elevated inflammatory index was associated with an increased risk of frailty. However, elevated inflammatory markers were not associated with an increased risk of having fair/poor HRQOL in AA candidates.

Our findings of higher levels of inflammatory markers among AAs compared to non-AAs are consistent with studies in community-dwelling adult populations [39, 40]. In the multi-ethnic population of the Dallas Heart Study, black subjects had higher CRP levels than white subjects [39], and as part of the Adventist Health Study-2, blacks had higher levels of CRP and IL6 compared to whites, yet no difference in TNFR1 levels. [40] Notably, we extend these community-dwelling studies of older adults (mean age 64–71) to ESRD patients across all ages and show that AAs have higher levels of TNFR1 and inflammatory index. The chronologic age of ESRD patients is likely not reflective of the true physiologic reserve you would see in community-dwelling patients of the same age. Thus, findings seen in older community-dwelling older adults are often seen in younger end-stage renal disease patients. We feel that ESRD does play a significant role in the accelerated aging process and puts these patients at increased risk of adverse health outcomes of aging.

Previous studies have shown greater survival of AAs on dialysis at higher levels of CRP [20] and IL-6 [21] which is consistent with our findings on the association between lower risk of adverse health outcomes and elevated inflammation in AAs compared to non-AAs. While inflammation, marked by elevated CRP and IL-6 levels, has been previously associated with frailty [11] and poorer physical function in older adults [15] our study extended these findings into a population of KT candidates, encompassing all ages. Our research builds on these previous findings and shows that elevated inflammation is an important measure to identify the risk of specific adverse health outcomes of aging such as frailty, SPPB impairment, and fair/poor HRQOL, all of which have been previously associated with poor outcomes including death and disability. Furthermore, we have shown that the impact of inflammation on adverse health outcomes of aging in KT candidates may be somewhat mitigated in AAs, and comorbidities in AAs may confound the association of inflammation and adverse health outcomes of aging. These findings are important to consider in the clinical practice and evaluation of end-stage renal disease patients showing that elevated inflammatory levels may lead to adverse outcomes in non-AAs and comorbidities in AAs may lead to more adverse outcomes rather than elevated inflammatory levels alone. Clinicians should consider measurement of inflammatory markers in addition traditional comorbidity assessment to identify who may benefit from prehabilitation or exercise training to prevent subsequent adverse health outcomes of aging [41].

This study has several important strengths, including a large sample size from two different hospitals, the measurement of the novel gerontology factor of frailty, lower extremity impairment, and fair/poor HRQOL (rather than the use of proxies), and the analysis of 3 inflammatory markers collected at the time of KT evaluation. Additionally, to preserve stored samples, we only analyzed the most recent serum samples (from 4/2014); those with and without analyzed blood samples only differed on smoking status. The blood draw included in this study was a cross-sectional representation on one day, so variability in inflammatory markers may be missed due to dialysis, infections, medications, or hospitalizations. This study has several limitations worth noting. This study is a cross-sectional analysis of inflammatory markers and adverse health outcomes of aging, so it is difficult to conclude a causal relationship between the exposure and outcome; unmeasured confounding is inherent in any observational study and may lead to inappropriate inferences. Additionally, we do not have information on the type of hemodialysis access (arteriovenous fistula, graft, or catheter) or the timing of lab draws relative to dialysis.

## Conclusions

We have shown that elevated inflammatory markers are associated with an increased risk of frailty, lower extremity impairment, and poor HRQOL among non-AAs with ESRD. While AAs experience an increased risk of frailty and lower extremity impairment with elevated inflammatory markers, non-AAs with elevated inflammatory markers had a higher risk of these adverse health outcomes of aging than non-AAs. This quantification provides early epidemiological evidence that the inflammatory pathway may be a novel and important target for reducing frailty burden and improving physical performance among KT candidates of all ages, especially among non-AAs. These findings highlight easy to measure markers of increased frailty, lower extremity impairment, and poor HRQOL risk and suggest that inflammatory markers should be measured during the evaluation process in order to identify individuals who may benefit from exercise training intervention or prehabilitation to improve their physical performance and lower their frailty burden while waiting for KT.

## Abbreviations

CCI: Charlson comorbidity index
CRP: C-reactive protein
ESRD: End-stage renal disease
HRQOL: Health-related quality of life
IL-6: Interleukin-6
KT: Kidney transplant(ation)
SPPB: Short physical performance battery
TNFR1: Tumor necrosis factor-α receptor-1
